# FunTaxIS-lite: a simple and light solution to investigate protein functions in all living organisms

**DOI:** 10.1093/bioinformatics/btad549

**Published:** 2023-09-06

**Authors:** Federico Bianca, Emilio Ispano, Ermanno Gazzola, Enrico Lavezzo, Paolo Fontana, Stefano Toppo

**Affiliations:** Computational Medicine Group (MedComp), Department of Molecular Medicine, University of Padova, Padova, Italy; Computational Medicine Group (MedComp), Department of Molecular Medicine, University of Padova, Padova, Italy; Computational Medicine Group (MedComp), Department of Molecular Medicine, University of Padova, Padova, Italy; Computational Medicine Group (MedComp), Department of Molecular Medicine, University of Padova, Padova, Italy; Research and Innovation Center, Edmund Mach Foundation, San Michele all'Adige, Trento, Italy; Computational Medicine Group (MedComp), Department of Molecular Medicine, University of Padova, Padova, Italy

## Abstract

**Motivation:**

Defining the full domain of protein functions belonging to an organism is a complex challenge that is due to the huge heterogeneity of the taxonomy, where single or small groups of species can bear unique functional characteristics. FunTaxIS-lite provides a solution to this challenge by determining taxon-based constraints on Gene Ontology (GO) terms, which specify the functions that an organism can or cannot perform. The tool employs a set of rules to generate and spread the constraints across both the taxon hierarchy and the GO graph.

**Results:**

The taxon-based constraints produced by FunTaxIS-lite extend those provided by the Gene Ontology Consortium by an average of 300%. The implementation of these rules significantly reduces errors in function predictions made by automatic algorithms and can assist in correcting inconsistent protein annotations in databases.

**Availability and implementation:**

FunTaxIS-lite is available on https://www.medcomp.medicina.unipd.it/funtaxis-lite and from https://github.com/MedCompUnipd/FunTaxIS-lite.

## 1 Introduction

The sequence data for many species has increased significantly due to the progress in omics sciences. However, characterizing the functional aspects of taxonomy poses substantial challenges. To address this, the Gene Ontology Consortium has collaborated internationally to provide ontologies describing unequivocally gene functions (GO terms) which are integrated in a direct acyclic graph. While GO-encoded annotations are independent of species, many of these annotations represent functions and biological processes that are not universally present across all taxa. The absence of explicit formalization regarding the specific protein functions that a particular species can exhibit (referred to as taxon constraints) can lead to improper functional transfers between proteins of different species, solely based on shared sequence similarities. Considering that protein/gene information in databases typically includes the species of origin, it becomes feasible to implicitly partition the GO graph based on taxonomic criteria. In this context, two types of taxonomic constraints can be identified: positive (“only-in-taxon”) and negative (“never-in-taxon”) relationships. Positive relationships define GO terms that can solely annotate a specific taxon, while negative relationships indicate GO terms that can never be applied to a taxon. The need to create taxonomic constraints appeared clear with the growth of the databases accumulating issues in the protein annotations due to the percolation of errors (Gilks *et al.* 2005). These problems persist not only in the protein annotations created automatically and labeled as “Inferred from Electronic Annotation” (IEA), which amount to 98% of the total annotations available, but also in manually curated protein functions. In GOA release of June 2023, for instance, “chloroplast thylakoid” (GO id: GO:0009534) is used to annotate two proteins Q38AK2 and Q4Q1E9 belonging to *Trypanosoma brucei brucei* and *Leishmania major*, respectively with the manually reviewed code Inferred from Biological aspect of Ancestor (IBA). These parasitic protozoans, that cause the sleeping sickness (*Trypanosoma*) and leishmaniasis (*Leishmania*) in humans, lack chloroplasts. For these reasons, since 2010, the Gene Ontology Consortium has developed a manually validated list of taxonomic constraints, specifying them at various levels of the ontology. However, manually defining constraints for over 40 000 GO terms and hundreds of thousands of species is a demanding task and present coverage of both species and GO terms is poor. To address this challenge, we have developed FunTaxIS-lite as an enhanced and faster version of its predecessor, FunTaxIS (Falda *et al.* 2016). FunTaxIS-lite enables the inference of functional peculiarities and similarities between different taxa, determining the presence or absence of specific functions in the vast majority of organism/species. The tool aims at: (i) being as broader as possible in covering the taxonomic tree and the GO graph, (ii) helping function prediction tools to discard wrong predicted annotations for the species under study, and (iii) helping database curators to fix potential issues of annotations in databanks.

## 2 FunTaxIS-lite pipeline

FunTaxIS-lite automatically assigns GO constraints to specific taxa and is able to integrate both the constraints defined by the GO Consortium (GOC) (Ashburner *et al.* 2000, The Gene Ontology Consortium 2021) and those manually curated by the user. This ensures a refined and tailored annotation process for exploring functional peculiarities across the taxonomic tree. The input files of the tool are the GOA database, the taxonomy database from NCBI, and the GO graph in Web Ontology Language (OWL) format. A breakdown of the main steps of the FunTaxIS-lite pipeline are herein explained (for further details, see Supplementary Material S1).

- Gene Otology Annotation (GOA) database cleaning step: GOA annotations undergo a cleaning process. The tool removes: annotations with no biological data available (“ND” evidence code), annotations stating that a protein does not perform a specific function (“NOT” evidence code), root GO terms (GO:0005575, GO:0008150, and GO:0003674) and annotations related to the tags “RNAcentral” and “environmental samples.”
- Taxonomic Reference Nodes determination: a list of highly annotated taxonomic reference node which are used to group organisms based on common biological traits, is established. The objective is to have a reliable set of constraints for each species subsumed by these reference taxa and to cover a broad range of the taxonomy hierarchy.
- Grouping GOs and cumulative frequencies calculation: GO annotations in the GOA database are grouped by organisms and, for each organism, a list of associated GO terms is generated. Each organism is traced back to its closest reference parent node. Then, for each GO term in the reference node, the cumulative frequency over its GO descendants is calculated.
- Creation of “never_in” GO Taxon Constraints: only GO terms with a cumulative frequency of at least 500 in the whole GOA database are considered. GO terms with a cumulative frequency of 0 in the reference taxon node are marked as “never_in” for that node and for all its descendant species. This means that the GO term (and all of its descendant GO terms in the GO graph) cannot be used to annotate any protein product in any species that belongs to the reference taxon node.
- Merging automatic, GOC, and manual constraints: in the final step, the automatic constraints generated by the FunTaxIS-lite program are combined with the GOC constraints, with the latter taking precedence in case of conflicts. The GOC constraints are defined as “never_in” or “only_in”, where “only_in” constraints specify that certain GO terms are restricted to a specific taxon. These constraints are converted into “never_in” for all of the species except those where the “only_in” is specified and added to the existing constraints. Moreover, a list of “manual constraints” is made based on direct observation of annotation issues that can be created by the end-user following a simple syntax in a configuration file. These constraints have the highest priority in the pipeline.

## 3 FunTaxIS-lite web server

FunTaxIS-lite is a freely accessible web site (https://www.medcomp.medicina.unipd.it/funtaxis-lite) and downloadable tool (https://github.com/MedCompUnipd/FunTaxIS-lite). Browsing the web site, the user can visualize and/or download the list of all prohibited GO terms (“never_in”) in tabular format by looking for taxa via taxonomic id or taxon name. FunTaxIS-lite tool can also be installed and run locally, allowing the user to explore different options and to add custom manual constraints to generate from scratch the lists of “never-in” terms. The parameters which can be customized are the database files from which the constraints are generated (GO graph file, GOA file, NCBI taxonomy files), the list of manual constraints and the cutoff on the cumulative frequency threshold.

## 4 FunTaxIS-lite vs FunTaxIS old version

FunTaxIS-lite is faster and more user-friendly than the previous version. One of the principal changes that differentiates it from FunTaxIS is the user’s capability to add the manually validated constraints to the automatically generated constraints, as well as those supplied by the GOC.

The main changes are:

- The number of “reference taxon nodes” has been expanded from 50 to 171 thanks to a new automated selection process based on the quantity and diversity of annotations at specific taxonomic nodes. The growth of the GOA dataset over time has also enabled this expansion of “reference taxon nodes.” The uneven distribution of annotations across taxa in the GOA has forced us to introduce two categories of reference taxonomic nodes: “Reliable” (70) and “Unreliable” (101). The “Reliable” nodes represent high-confidence nodes in the taxonomic hierarchy that group well-annotated branches. They owe their robustness to the inclusion of extensively studied model organisms with rich functional information. On the other hand, the “Unreliable” nodes group poorly annotated taxonomic branches with limited available knowledge for which the tool is able to define a small but usable set of constraints.
- Simplification of calculations. Within the tool, we have made the selection of “never_in” GO terms more efficient by removing the previous fuzzy logic that didn’t provide any effective contribution.
- Completely revisited the integration of GOC constraints due to the introduction of the novel manual constraints and resolution of potential conflicts among manual and automatic constraints.
- A more user-friendly, light, and effective web site has been developed, offering rapid access to the “never_in” GO list corresponding to any species within the taxonomic tree. All the taxon-constraints are precomputed and loaded into the database, which is periodically updated by our research group, according to the latest releases of GO, GOA, and Taxonomy databases.

## 5 Benchmark datasets and assessment

The “functional domain” created by FunTaxIS-lite refers to a collection of Gene Ontology (GO) terms that establish the defined boundaries within which the species can exert its own functions. We performed a “species-centric” assessment by designing a benchmark where a similarity search based on DIAMOND (Buchfink *et al.* 2015) and our automatic function prediction tool Argot (Lavezzo *et al.* 2016) have been challenged to annotate the proteins coming from four different species in a classical blind test. The four species have been chosen as representatives of highly different taxonomic ranks: the plant *Amborella trichopoda* (tax id: 13333), the lobe-fin bony fish *Latimeria chalumnae* (tax id: 7897), the bacterium *Pseudomonas fluorescens SBW25* (tax id: 216595), and the yeast *S. kudriavzevii IFO 1802* (tax id: 226230). UniProt protein database (2023_03) has been used for the similarity searches (DIAMOND) and Argot predictions. Proteins attributed to the taxonomic group Bacteria have been excluded from UniProt for *P.fluorescens*, whereas proteins associated with the taxon node Eukaryotes have been eliminated for *A.trichopoda*, *S.kudriavzevii IFO 1802*, and *L.chalumnae* before using Argot and DIAMOND. This strategy has been chosen to put DIAMOND and Argot methods under probative test conditions where only distantly related species are available in the protein databank. Annotations obtained from these two methods were filtered using the constraints generated by FunTaxIS-lite and by PANNZER (Törönen and Holm 2022), for comparative purposes. The “species-centric” benchmark involves aggregating all the predicted annotations obtained from any protein belonging to the species under evaluation in a single non-redundant set of GO terms. Since a GO term can be associated with multiple target proteins, its score has been defined as the highest score obtained by the prediction tool for that particular GO term. This ensures that the highest confidence is considered for every function attributed to a certain species, while eliminating GO term redundancy. To evaluate the performance, we used the official metrics used in the Critical Assessment of protein Function Annotation challenge (CAFA) (Zhou *et al.* 2019): F_max_ that is the harmonic mean of precision and recall measures, the weighted F_max_ (wF_max_) that considers the information content (*ic*) of each GO term to calculate the F_max_ and the minimum semantic distance (S_min_) from the remaining uncertainty (*ru*) and misinformation (*mi*) (Jiang *et al.* 2016). The details of the metrics are reported in Supplementary Material S1. We have evaluated the metrics for each subontology of GO: Biological Process (BP), Cellular Component (CC), and Molecular Function (MF).

## 6 Results

The constraints generated by FunTaxIS-lite encompass not only its own constraints but also those from GOC. We have conducted a thorough investigation to determine the extent of our automatic taxonomic constraints compared to those provided by GOC, and the differences between them are minimal. The majority of the manually curated GOC constraints are also present in our tool alone. Moreover, our approach, which relies on propagation rules over both the taxonomy and the GO graph, along with the extensive coverage of the taxonomic hierarchy, has enabled us to expand the GOC constraints by an average of 300% in every taxon node considered by GOC (see Supplementary Table S1).

**Figure 1.**
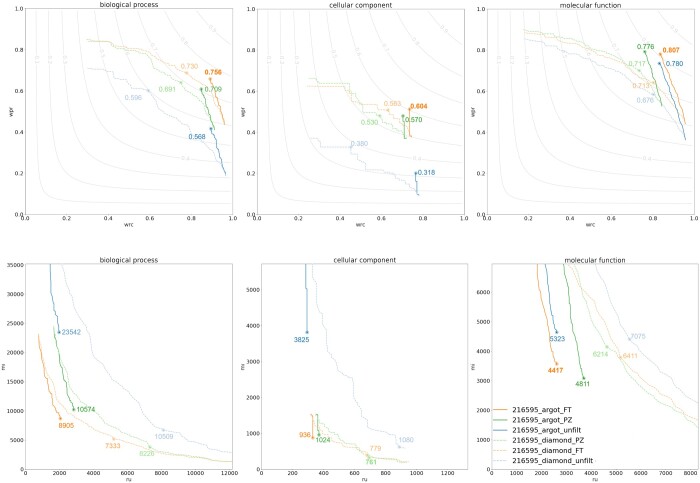
Comparison of evaluation metrics of FunTaxIS-lite and PANNZER. (A) This panel shows the performances for the *species P.fluorescens SBW25* (tax ID: 216595). We have evaluated wF_max_ for each subontology (BP, CC, and MF) starting from the GO terms extracted by the protein hits found by DIAMOND (dashed line) and Argot (solid line). Performances have been evaluated without filtering (unfilt) and using both FunTaxIS-lite (FT) and PANNZER (PZ) taxonomic constraints. In Panel B, the S_min_ evaluation is reported for each subontology.

When compared with its predecessor version, FunTaxIS-lite always performs better in all the tested scenarios (Table 1) and in any metrics used (see also Supplementary Table S2). The detailed comparison of taxonomic filters applied to DIAMOND and Argot results is presented in Fig. 1 for *P.fluorescens SBW25*. The results for *A.trichopoda*, *L.chalumnae*, and *S.kudriavzevii IFO 1802* are available in Supplementary Material. The baseline is established by plotting the unfiltered set of GO terms from both Argot and DIAMOND, providing a reference to evaluate the improvements achieved by applying the FunTaxIS-lite and PANNZER filters. The combination of Argot and FunTaxIS-lite filters (argot_FT) demonstrates superior performance in each subontology, as measured by F_max_ and wF_max_. For the best S_min_ metric, the performance of the DIAMOND + FunTaxIS-lite filters (diamond_FT) is slightly better in BP, while the DIAMOND + PANNZER filters (diamond_PZ) outperform in CC, and Argot + FunTaxIS-lite filters perform better in MF. In general, FunTaxIS-lite performs well across all species used in this study, enhancing the assessment metrics of both DIAMOND and ARGOT unfiltered predictions on average, respectively, by 8.32% and 5.88% for *A.trichopoda*; by 8.63% and 5.58% for *L. calumniae*; by 22.21% and 42.08% for *P.fluorescens SBW25*; by 7.22% and 2.64% for *S. kudriazevii IFO 1802* (for more detailed results see Supplementary Table S2). Performances measured with FunTaxIS-lite filters are overall better than those with PANNZER filters except few cases. PANNZER shows a slight advantage over both wF_max_ and S_min_ for CC in *P.fluorescens SBW25* and *L.calumniae*. This could be attributed to our strategy, which considers deeper taxonomic nodes compared to PANNZER, enabling the inclusion of IEA annotations in our lists. Further analysis of this effect is presented in Section 7.

**Table 1.** Comparative analysis of FunTaxIS-lite and FunTaxIS.^a^

Species	Ns	Tool	F_max_	wF_max_	S_min_
***A.trichopoda***	**BP**	FT_lite	**0.665**	**0.591**	**37** **367**
		FT_old	0.633	0.562	38 003
	**MF**	FT_lite	**0.778**	**0.711**	**10** **801**
		FT_old	0.733	0.664	12 421
	**CC**	FT_lite	**0.499**	**0.417**	**7171**
		FT_old	0.485	0.406	7187
***L.chalumnae***	**BP**	FT_lite	**0.555**	**0.483**	**46** **399**
		FT_old	0.537	0.467	46 945
	**MF**	FT_lite	**0.764**	**0.707**	**9736**
		FT_old	0.718	0.659	11 148
	**CC**	FT_lite	**0.522**	**0.456**	**5830**
		FT_old	0.518	0.453	5832
***P.fluorescens SBW25***	**BP**	FT_lite	**0.801**	**0.757**	**8905**
		FT_old	0.702	0.64	16 887
	**MF**	FT_lite	**0.845**	**0.807**	**4417**
		FT_old	0.823	0.781	5302
	**CC**	FT_lite	**0.668**	**0.604**	**936**
		FT_old	0.508	0.422	2298
***S.kudriavzevii IFO 1802***	**BP**	FT_lite	**0.778**	**0.732**	**8559**
		FT_old	0.773	0.727	8633
	**MF**	FT_lite	**0.822**	**0.784**	**3415**
		FT_old	0.815	0.776	3534
	**CC**	FT_lite	**0.559**	**0.499**	**3077**
		FT_old	0.557	0.497	3080

a The table shows the performance comparison between FunTaxIS-lite (FT_lite) and the previous version (FT_old). The results for *A.trichopoda*, *L.chalumnae*, *P.fluorescens SBW25*, and *S.kudriavzevii IFO 1802* are reported. The metrics F_max_, wF_max_, and S_min_ have been calculated for the BP, MF, and CC subontologies for each species. The best performing filter between FunTaxIS-lite and old FunTaxIS is highlighted in bold. The table exclusively presents Argot results as the performances of DIAMOND are found to be identical.

## 7 Discussion

The process of functional annotation of genes and proteins largely depends on automated tools (over 98% of annotations are IEA), which perform functional transfer between highly (and experimentally) studied organisms and other, less known, species. This process must be tightly regulated, to avoid the erroneous transfer of functions that are not present in the functional domain of the recipient organism. On the other hand, if functional transfer between species is not allowed, the annotation coverage of millions of proteins present in UniProt databank would be marginal. The generation of functional constraints for the entire taxonomy is a challenging task, particularly due to biases inherent in functional databases and the limited availability of experimental studies. To ensure a wide coverage of the constraints over the taxonomy, the exploitation of experimental annotations alone is not sufficient, making it necessary to rely also on electronically inferred functions. Additionally, experimental annotations are not always reliable, as we pointed out with some examples in the introduction section to achieve this accomplishment, we have developed FunTaxIS-lite, which provides information about allowed/forbidden annotations at the species level for almost the entire taxonomy. To mitigate the biases of functional databases, we have employed two complementary strategies. Firstly, we have considered as many model organisms as possible, thereby increasing the granularity of the functional domains. Secondly, we have restricted the generation of constraints to functions that are adequately represented in GOA, excluding highly specific functions that may be associated exclusively with a single model organism. The significant difference in the number of “never_in” constraints provided by FunTaxIS-lite compared to GOC is noteworthy. This outcome is a direct result of the strategic approach we have implemented, which involves exploring both the taxonomy and the Gene Ontology (GO) graph. As a result, we not only obtain more fine-grained constraint lists but also capture a larger amount of information embedded in the databases.

The improvement in prediction performance when filters are applied shows a great difference between *P.fluorescens SBW25* and all other species. After careful investigation, we have identified two main factors that are responsible for this effect. Firstly, the benchmarking approach involved excluding all proteins related to *Bacteria* and its taxonomic descendants from the database during the prediction process, which led to a much higher number of incorrect annotations (False Positives hits) compared to the other three species. In addition, generating “never-in” filters for bacterial species has proven to be less challenging than for other taxonomic nodes due to the reduced functional variability within the taxonomic node *Bacteria*. The combined effect of these two factors, (i) high quality filters and (ii) predictions with a very high background noise, has contributed significantly to the marked increase in prediction performance for *P.fluorescens SBW25* compared to the other three species. This has been observed both for FunTaxIS-lite and PANNZER filters.

In comparison with PANNZER, which is another tool with similar purposes, FunTaxIS-lite displays better performance on most of the tested settings, with differences that are likely due to the different logical approaches of the two algorithms. FunTaxIS-lite defines a list of prohibited GO terms for many different nodes across the taxonomy, while PANNZER define the GO terms that are allowed for only highly generic taxon nodes and inherited by all their descendants. This approach, although valid in many cases, can sometimes lead to wrong outcomes. To make an example, we have found that some GO terms predicted for the species evaluated in the benchmark were not purged by the PANNZER filters. For example, both Argot and DIAMOND erroneously reported the GO term “GO:0009521” photosystem for the species *P.fluorescens SBW25*. This GO term is correct for some prokaryotes (*cyanobacteria*, for example), but this is not true for *Pseudomonadales*, belonging to a completely different branch of prokaryotes. The PANNZER filters are also too stringent in a few cases, as they purge correct GO terms from the ground truth. For example, the term “GO:0006147” guanine catabolic process is vastly present in many bacteria, including *Pseudomonadales*, as IEA annotations (>25 000) and is thus reasonably associated with bacteria, but disallowed by PANNZER non-IEA based filters.

## 8 Conclusions

To summarize, the taxonomic filters generated by FunTaxIS-lite are useful for both curators and biologists. They can be used in different scenarios. (i) Investigating specific functions oddly absent in some taxa. (ii) Spotting possible errors in the database. If a particular function is reported for a taxon, but it is not allowed by the taxonomic filters, then it is possible that the annotation is incorrect. (iii) Refining the output of automatic protein function prediction tools. Automatic protein function prediction tools use a variety of sources of information to predict the functions of proteins. However, these tools can sometimes make mistakes. By using the taxonomic filters, it is possible to remove some false positive annotations from the output of these tools, which can improve the accuracy of the predictions. As shown in the results comparing the unfiltered versus filtered results of Argot, the taxonomic filters can significantly improve the accuracy of automatic protein function prediction tools.

## Supplementary Material

btad549_Supplementary_Data

## Data Availability

FunTaxIS-lite is available on https://www.medcomp.medicina.unipd.it/funtaxis-lite and from https://github.com/MedCompUnipd/FunTaxIS-lite.
