# Supplementary material for: FunTaxIS-lite: a simple and light solution to investigate protein functions in all living organisms
Source: Bioinformatics. 2023 Sep 6;39(9):btad549. doi: 10.1093/bioinformatics/btad549 (PMC10500080; doi:10.1093/bioinformatics/btad549)
Supplement: btad549_Supplementary_Data [file btad549_supplementary_data.zip › S1.docx]

**FunTaxIS-lite: a simple and light solution to investigate protein functions in all living organisms**

Federico Bianca^1 $^, Emilio Ispano^1 $^, Ermanno Gazzola^1 $^, Enrico Lavezzo^1^, Paolo Fontana^2^, Stefano Toppo^1^.

^1^Computational Medicine Group (MedComp), Department of Molecular Medicine, University of Padova, via Cristoforo Colombo 2, Padova, Italy.

^2^Research and Innovation Center, Edmund Mach Foundation, San Michele all'Adige, Trento, Italy, ^$^Equal contribution to work.

**Availability:** FunTaxIS-lite is available on <https://www.medcomp.medicina.unipd.it/funtaxis-lite> and from https://github.com/MedCompUnipd/FunTaxIS-lite.

**Contacts**: stefano.toppo@unipd.it.

Summary

[SUPPLEMENT 1: FunTaxIS-lite working pipeline 3](#_Toc141350940)

[1.1 GOA database cleaning 3](#_Toc141350941)

[1.2 Taxonomic Reference Nodes determination 4](#_Toc141350942)

[1.3 Grouping GOs and cumulative frequencies calculation 4](#_Toc141350943)

[1.4 Creation of “never in” GO Taxon Constraints 4](#_Toc141350944)

[1.5 Merging automatic, consortium, and manual constraints 6](#_Toc141350945)

[SUPPLEMENT 2: Benchmarking 7](#_Toc141350946)

[2.1 Evaluation metrics 7](#_Toc141350947)

[2.2 Results regarding all species for the comparison between FunTaxIS-lite and PANNZER 8](#_Toc141350948)

[SUPPLEMENT 3: Comparison between automatic constraints (FunTaxIS-lite) and consortium constraints (GOC). 12](#_Toc141350949)

# SUPPLEMENT 1: FunTaxIS-lite working pipeline

The following sections describe each step of the FunTaxIS-lite pipeline. A brief scheme is shown in **Figure S1a**.


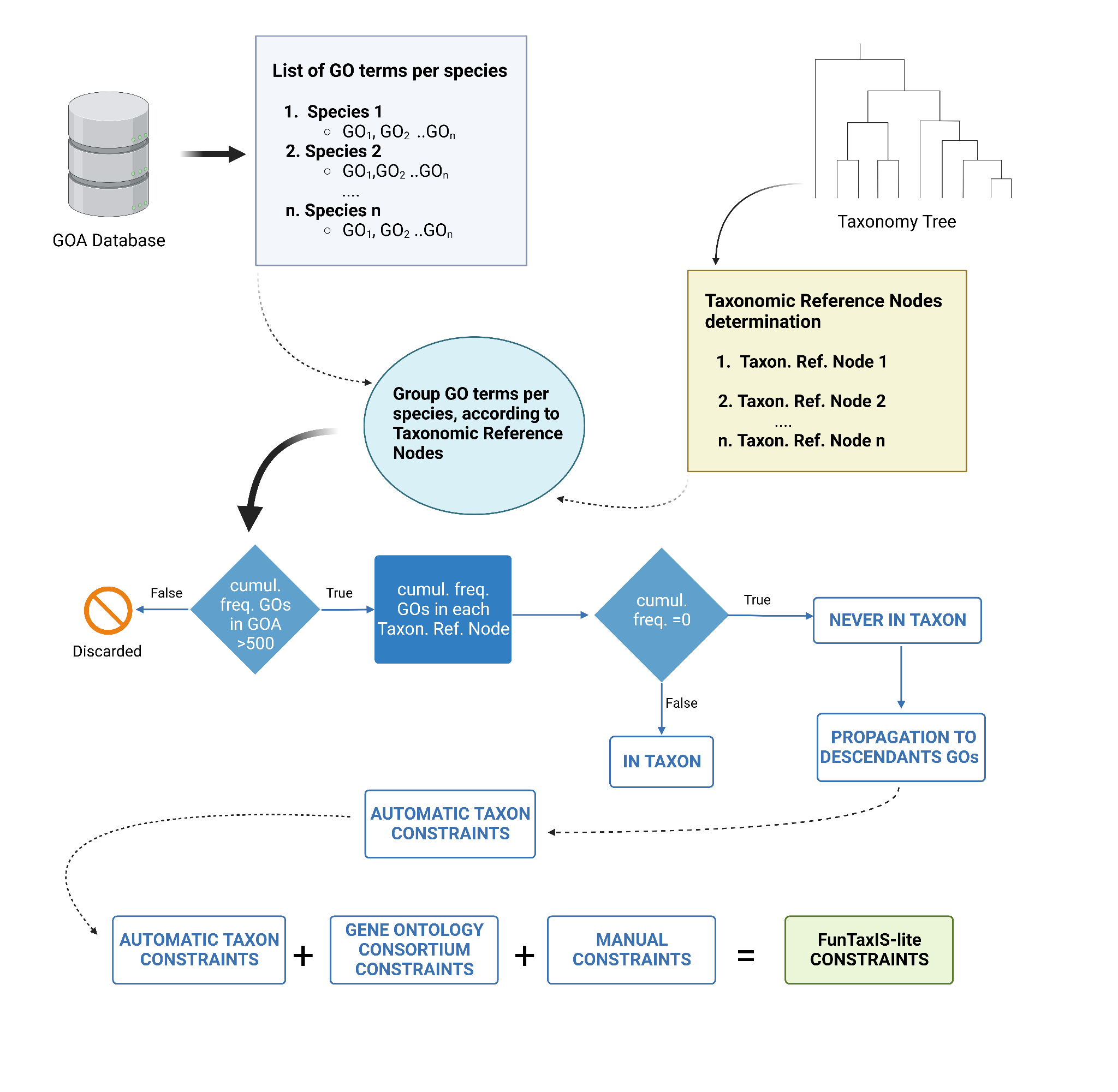


***Figure S1a:*** *Schematic representation of the FunTaxIS-lite pipeline. The picture shows the different steps necessary to generate the taxon constraints.*

## 1.1 GOA database cleaning

The first step requires a cleaning process of the raw Gene Ontology Annotation (GOA), provided as a GO Annotated File (GAF) format by the Gene Ontology Consortium (GOC). The cleaning step is required to remove either uninformative or peculiar data. For instance, annotations with the curator evidence code “ND” annotations (No biological Data available), with “NOT” qualifier (protein does NOT perform a specific function), root GO terms (GO:0005575, GO:0008150 and GO:0003674), and annotations with tag “RNAcentral” and “environmental samples” are removed from the database.

## 1.2 Taxonomic Reference Nodes determination

A pivotal step in the FunTaxIS-lite pipeline is to determine a list of taxonomic reference nodes to be used to group organisms and their respective annotation contribution, which share common biological features. For each reference taxonomic node, a list of taxonomic constraints is then produced and inherited by all organisms subsumed by that reference taxonomic node.

Our approach to identify reference taxonomic nodes for functional constraints involves balancing two objectives:

1. having a reliable set of constraints for each species subsumed by the corresponding reference taxon;
2. covering a broad range of the taxonomy hierarchy.

This resulted in a total of 171 reference taxonomic nodes representative of all the metabolic/signaling pathways performed by the organisms they subsume. For example, it is crucial to have a solid list of disallowed functions (e.g. photosynthesis for animals or nervous system for plants). However, due to the inhomogeneity of distribution of annotations in the GOA, the annotation coverage varies widely among taxonomic ranks, leading to two categories of reference taxonomic nodes:

1) "Reliable taxonomic nodes" are highly representative nodes in the taxonomy hierarchy that group well-annotated branches thanks to the presence of model organisms that are extensively studied and rich in functional features.

2) "Unreliable taxonomic nodes" are generic nodes in the taxonomy hierarchy and group poorly annotated branches with limited available knowledge to generate a strong set of constraints.

## 1.3 Grouping GOs and cumulative frequencies calculation

The annotations present in the GOA database are grouped by organisms and for each of them the list of all the associated GO terms is produced, including details about the frequency, evidence code, and ontology. All species and their annotations are traced back to their “closest” reference parent node. Subsequently, for each GO term of the reference node, the cumulative frequency over its descendants is calculated.

## 1.4 Creation of “never in” GO Taxon Constraints

Once the cumulative frequency of each GO term is obtained for every reference taxon node, the “never-in” constraints are generated following two main steps:

- **Cut-off 500**: Only GO terms with a cumulative frequency >= 500 in the whole GOA are considered. Finally, a GO term for which the cumulative frequency in a reference node is 0 is tagged as “never-in” for that reference node. **Figure S1b** provides an illustration of how the cumulative frequency of each GO term for each species contributes to its taxonomic reference node.


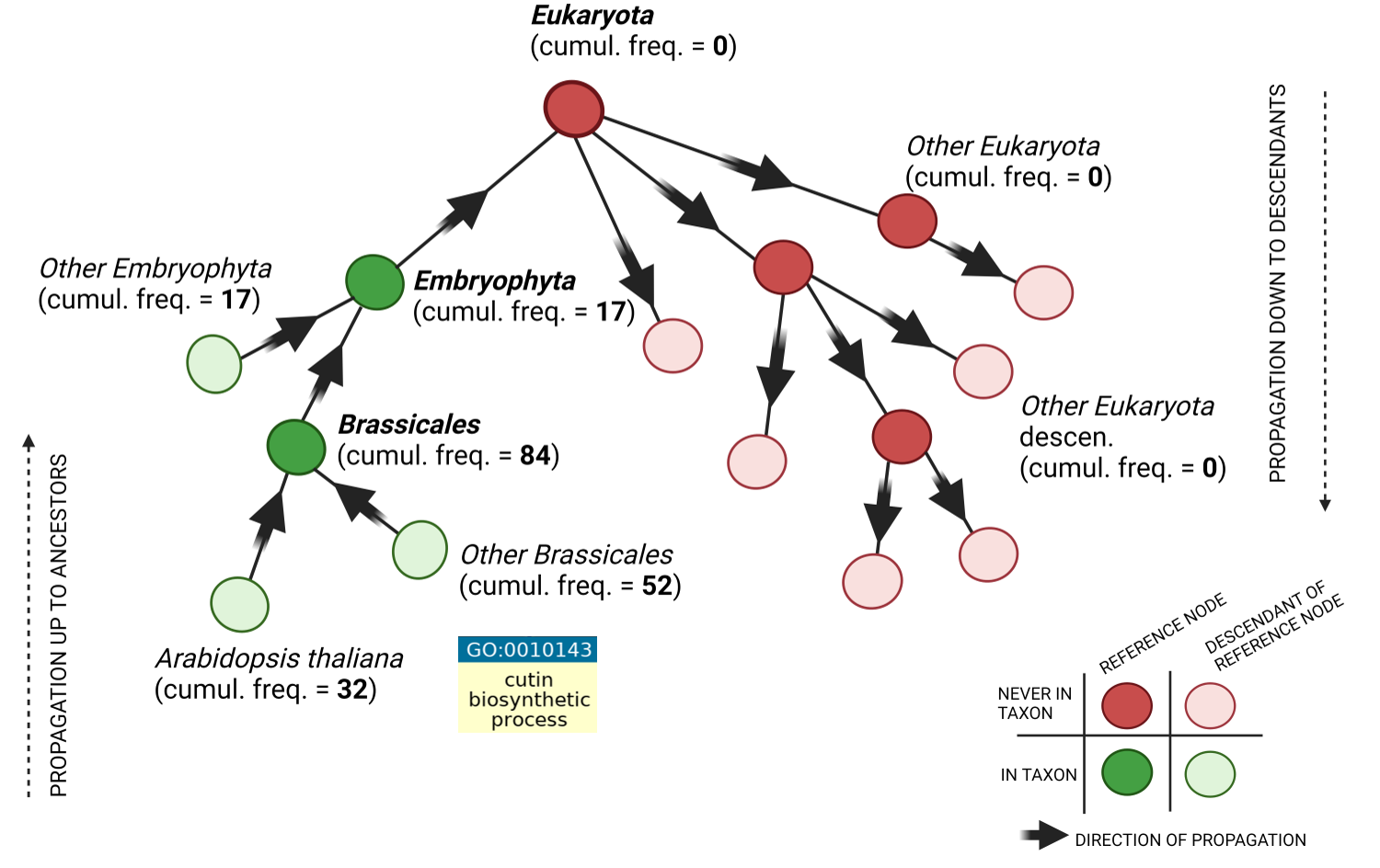


***Figure S1b****: The figure shows an example for the GO term GO:0010143 “cutin biosynthetic process”. This term is allowed for plant species since its cumulative frequency is above 0 for both Brassicales and Embryophyta, while it is forbidden for other Eukaryotes since the frequency is 0 for Eukaryota reference node. The figure also shows that annotations coming from the model organism Arabidopsis thaliana contribute to its “closest” reference node (Brassicales) whilst they do not contribute to the upper node Embryophyta that instead receives annotations from other organisms.*

- **Propagation**: the “never-in” generated in the previous step are then propagated down the GO graph following the “true path rule” that governs the graph, i.e. if a parent node is “false” then its child nodes inherit the ”false” property. As a result, GO terms that may have been discarded by the aforementioned cut-off of 500 could be recovered as “never-in” for that taxonomic reference node (**Figure S1c**).


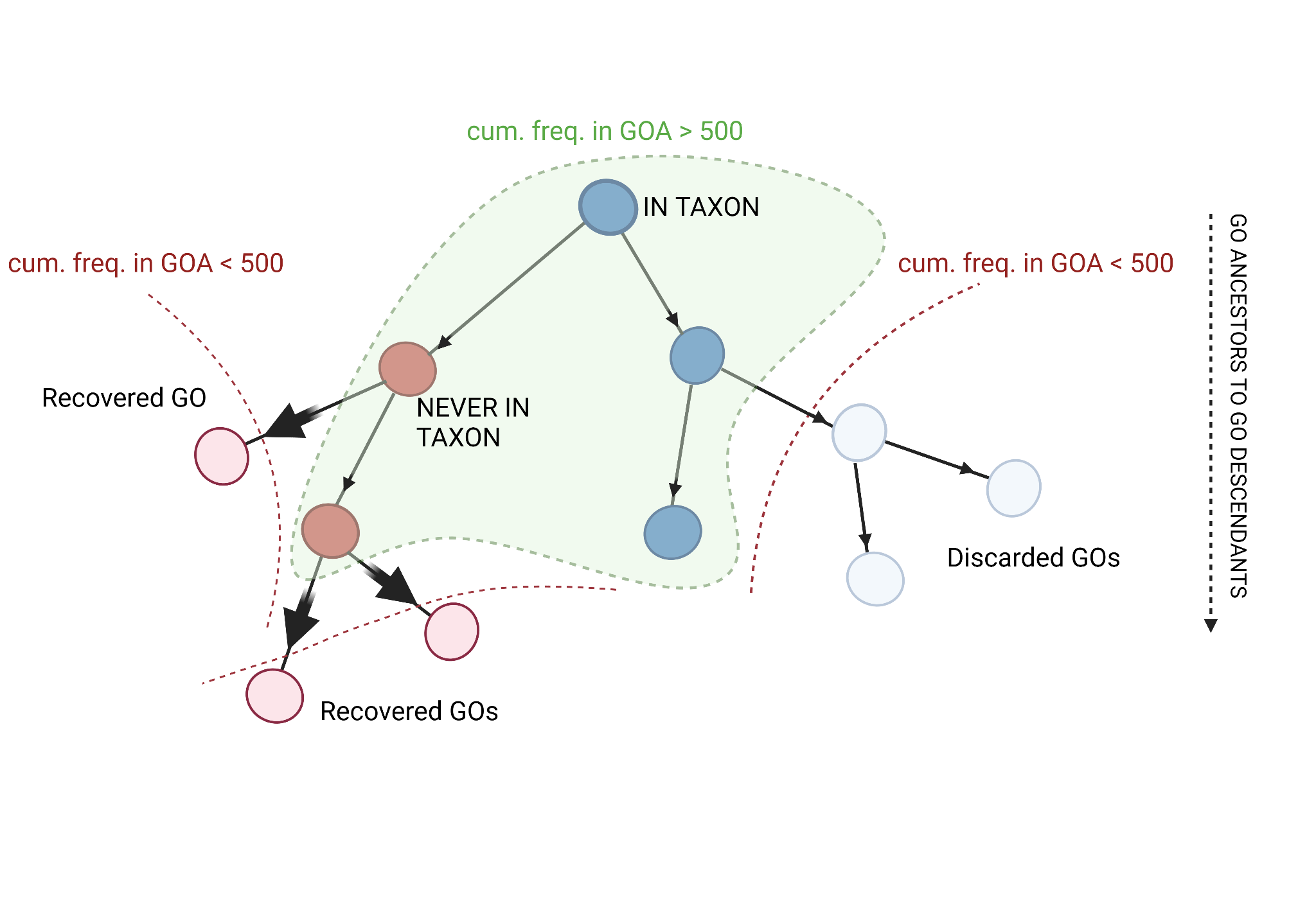


***Figure S1c****: Propagation of “never-in” over the GO graph. The "true path rule" that governs the GO graph dictates that properties, such as "never-in" constraints, are propagated downstream through the graph. In the figure, cyan and light blue nodes report terms that are “in-taxon” while red ones indicate “never-in-taxon” terms. In particular, light red nodes with a cumulative frequency in GOA < 500 inherit the property from red nodes that have a cumulative frequency > 500. As a result, many GO terms that were not initially considered are now recovered and then considered for the creation of taxonomic constraints.*

## 1.5 Merging automatic, consortium, and manual constraints

In the final step of FunTaxIS-lite, the automatic constraints generated by the program are combined with those provided by the Gene Ontology Consortium (referred to as "consortium constraints"). The consortium constraints take precedence over the automatic ones and can override them. Unlike the constraints generated by FunTaxIS-lite, which are only "never-in", the consortium can also provide the "only-in" constraints. "only-in" constraints can contain additional information, as they can be used to annotate only organisms from a specific taxon, which automatically makes these GO terms "never-in" for all other species in the taxonomy tree. As a result, the "only-in" constraints are converted into "never-in" and added to the existing constraints generated by FunTaxIS-lite to increase the total number of constraints. Additionally, to correct errors caused by incorrect annotations in the GOA, a brief list of "manual constraints" is created based on first-hand observation of annotation issues. These constraints are given the highest priority. In summary, the tool assigns priorities to the different types of constraints as follows: "manual constraints" have the highest priority, followed by "consortium constraints", and finally, "automatic constraints" have the lowest priority.

# SUPPLEMENT 2: Benchmarking

## 2.1 Evaluation metrics

The performances of the methods have been evaluated following standard evaluation metrics of the CAFA challenge that are hereby presented extensively. True positives $TP\left( \tau\right)\left( g \right)$, False negatives $FN\left( \tau\right)\left( g \right)$ and False positives $FP\left( \tau\right)\left( g \right)$ ratios are calculated for each GO term *g*:

$$TP\left( \tau\right)\left( g \right)=\left( g\in P\left( \tau\right)\cap g\in T \right)$$

$$FN\left( \tau\right)\left( g \right)=\left( g\notin P\left( \tau\right)\cap g\in T \right)$$

$$FP\left( \tau\right)\left( g \right)=\left( g\in P\left( \tau\right)\cap g\notin T \right)$$

Where $\tau$ is the value of the mobile threshold at which the ratios are calculated, $P\left( \tau\right)$ the set of predicted terms above the threshold value and $T$ the set of terms present in groundtruth. From this, precision $pr\left( \tau\right)$*,* recall $rc\left( \tau\right)$ and their harmonic mean$F_{max}$ are calculated as follows, where $\mathbb{1}$ () is an indicator function that returns either 1 or 0 if its conditions are met, namely if the term *g* is a TP, FN or FP where demanded or not:

$$pr\left( \tau\right)=\frac{\sum_{g} \mathbb{1}\left( TP\left( \tau\right)\left( g \right) \right)}{\sum_{g} \left( \mathbb{1}\left( TP\left( \tau\right)\left( g \right) \right)\mathbb{+1}\left( FP\left( \tau\right)\left( g \right) \right) \right)}$$

$$rc\left( \tau\right)=\frac{\sum_{g} \mathbb{1}\left( TP\left( \tau\right)\left( g \right) \right)}{\sum_{g} \left( \mathbb{1}\left( TP\left( \tau\right)\left( g \right) \right)\mathbb{+1}\left( FN\left( \tau\right)\left( g \right) \right) \right)}$$

$$F_{max}={max}_{\tau}\left( 2\cdot\frac{pr\left( \tau\right)\cdot rc\left( \tau\right)}{pr\left( \tau\right)+rc\left( \tau\right)} \right)$$

We decided also to exploit alternative versions of these formulas weighted around the information content $ic\left( g \right)$ of each considered term. The information content of a given GO term *g* is computed as follows, where $f_{c}\left( g \right)$ stands for the cumulative frequency of the term, calculated over its descendants, and $f_{c}\left( root \right)$ that of the corresponding root ontology term:

$$ic\left( g \right)=-log \frac{f_{c}\left( g \right)}{f_{c}\left( root \right)}$$

From this, the weighted precision $wpr\left( \tau\right)$, recall $wrc\left( \tau\right)$ and $wF_{max}$ are calculated:

$$wpr\left( \tau\right)=\frac{\sum_{g} ic\left( g \right)\mathbb{\cdot1}\left( TP\left( \tau\right)\left( g \right) \right)}{\sum_{g} ic\left( g \right)\cdot\left( \mathbb{1}\left( TP\left( \tau\right)\left( g \right) \right)\mathbb{+1}\left( FP\left( \tau\right)\left( g \right) \right) \right)}$$

$$wrc\left( \tau\right)=\frac{\sum_{g} ic\left( g \right)\mathbb{\cdot1}\left( TP\left( \tau\right)\left( g \right) \right)}{\sum_{g} ic\left( g \right)\cdot\left( \mathbb{1}\left( TP\left( \tau\right)\left( g \right) \right)\mathbb{+1}\left( FN\left( \tau\right)\left( g \right) \right) \right)}$$

$$wF_{max}={max}_{\tau}\left( 2\cdot\frac{wpr\left( \tau\right)\cdot wrc\left( \tau\right)}{wpr\left( \tau\right)+wrc\left( \tau\right)} \right)$$

Additionally, we also computed the minimum semantic distance $S_{min}$ from its composing metrics misinformation $mi\left( \tau\right)$ and remaining uncertainty $ru\left( \tau\right)$:

$$mi\left( \tau\right)=\sum_{g} ic\left( g \right)\mathbb{\cdot1}\left( FP\left( \tau\right)\left( g \right) \right)$$

$$ru\left( \tau\right)=\sum_{g} ic\left( g \right)\mathbb{\cdot1}\left( FN\left( \tau\right)\left( g \right) \right)$$

$$S_{min}={min}_{\tau}\left( \sqrt{{mi\left( \tau\right)}^{2}+{ru\left( \tau\right)}^{2}} \right)$$

## 2.2 Results regarding all species for the comparison between FunTaxIS-lite and PANNZER filters


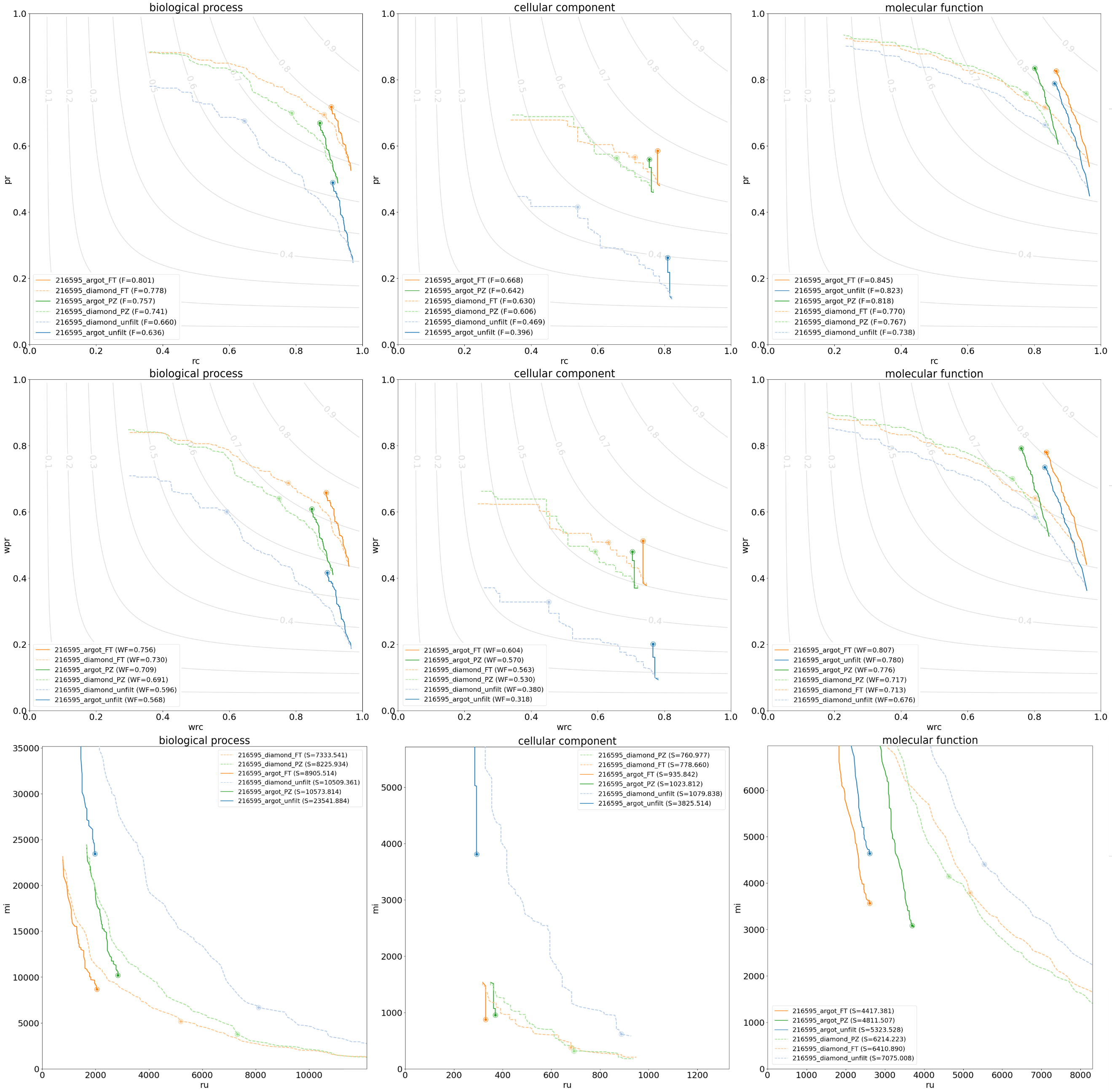


***Figure S2.1:*** *Comparison of evaluation metrics of FunTaxIS-lite and PANNZER. Panel A) shows the performances for the species P. fluorescens SBW25 (tax ID: 216595). We have evaluated Fmax for each subontology (BP, CC, and MF) starting from the GO terms extracted by the protein hits found by DIAMOND (dashed line) and Argot (solid line). Performances have been evaluated without filtering (unfilt, blue line) and using both FunTaxIS-lite (FT, orange line) and PANNZER (PZ, green line) taxonomic constraints. In panel B), the wFmax evaluation is reported for each subontology. In panel C), the Smin evaluation is reported for each subontology.*

C)

B)

A)


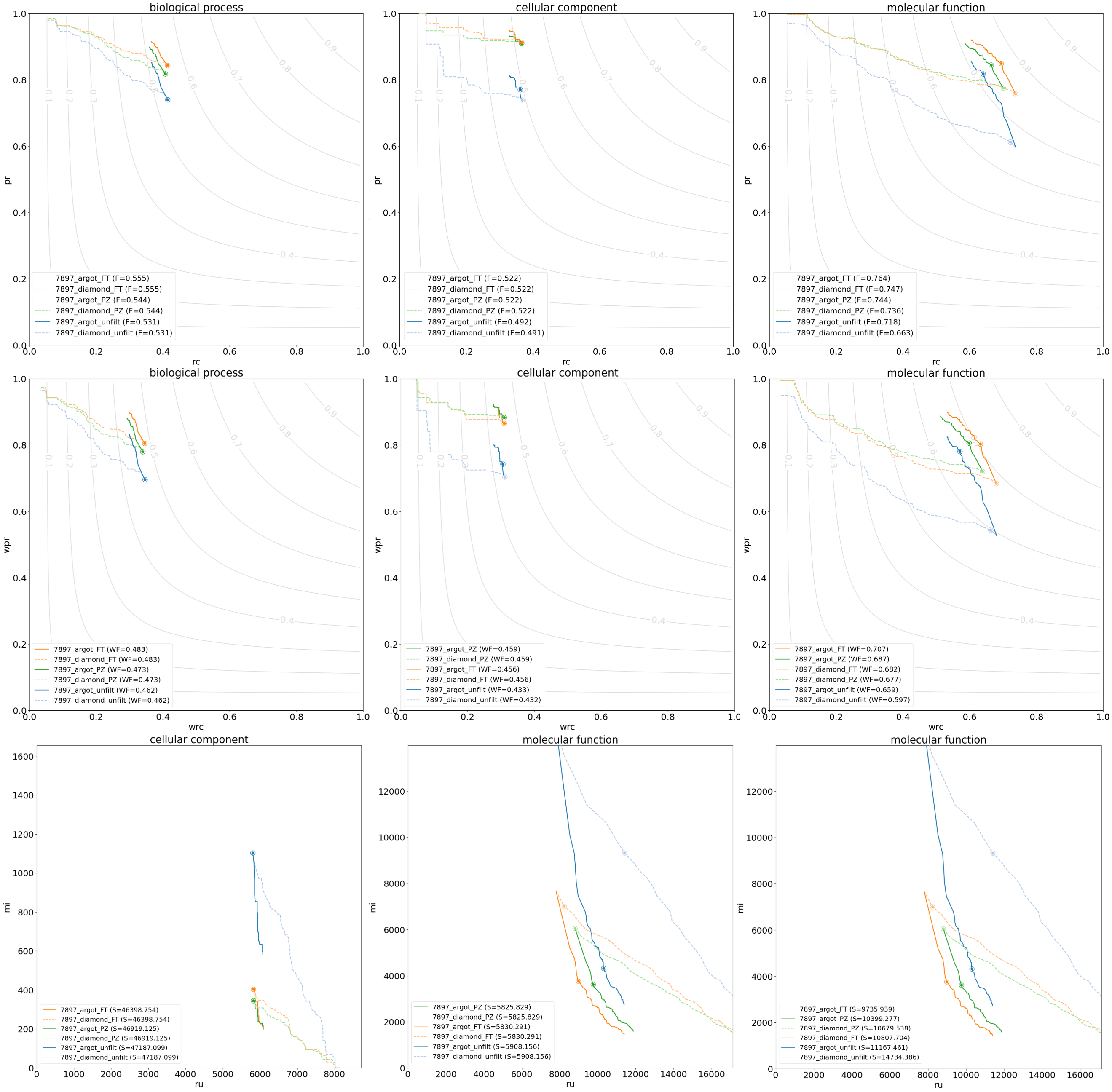


C)

B)

A)

***Figure S2.2:*** *Comparison of evaluation metrics of FunTaxIS-lite and PANNZER. Panel A) shows the performances for the species L. chalumnae (tax ID: 7897). We have evaluated Fmax for each subontology (BP, CC, and MF) starting from the GO terms extracted by the protein hits found by DIAMOND (dashed line) and Argot (solid line). Performances have been evaluated without filtering (unfilt, blue line) and using both FunTaxIS-lite (FT, orange line) and PANNZER (PZ, green line) taxonomic constraints. In panel B), the wFmax evaluation is reported for each subontology. In panel C), the Smin evaluation is reported for each subontology.*


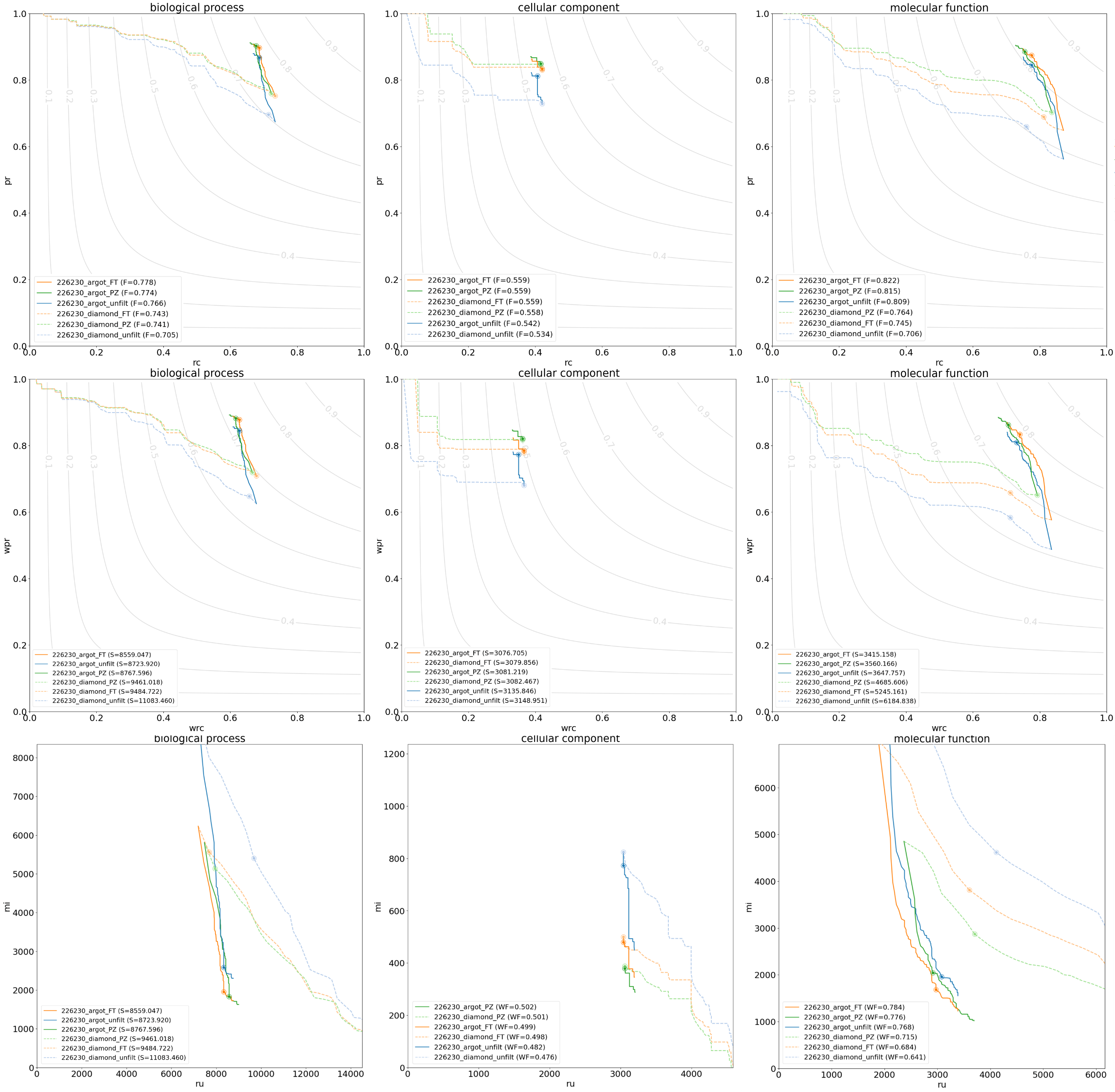


A)

B)

C)

***Figure S2.3:*** *Comparison of evaluation metrics of FunTaxIS-lite and PANNZER. Panel A) shows the performances for the species S. kudriavzevii IFO 1802 (tax ID: 226230). We have evaluated Fmax for each subontology (BP, CC, and MF) starting from the GO terms extracted by the protein hits found by DIAMOND (dashed line) and Argot (solid line). Performances have been evaluated without filtering (unfilt, blue line) and using both FunTaxIS-lite (FT, orange line) and PANNZER (PZ, green line) taxonomic constraints. In panel B), the wFmax evaluation is reported for each subontology. In panel C), the Smin evaluation is reported for each subontology.*


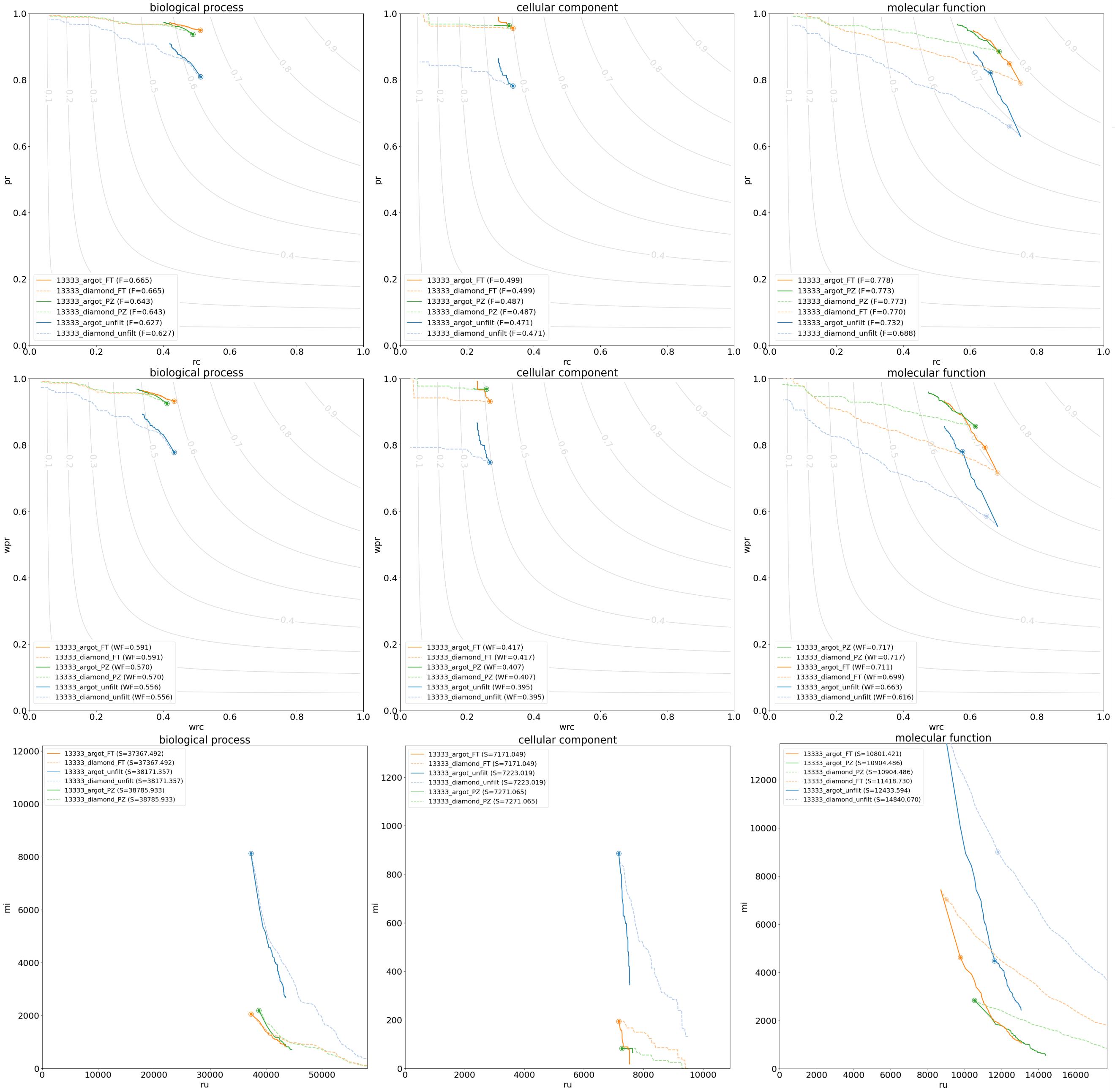


C)

B)

A)

***Figure S2.4:*** *Comparison of evaluation metrics of FunTaxIS-lite and PANNZER. Panel A) shows the performances for the species A. trichopoda (tax ID: 13333). We have evaluated Fmax for each subontology (BP, CC, and MF) starting from the GO terms extracted by the protein hits found by DIAMOND (dashed line) and Argot (solid line). Performances have been evaluated without filtering (unfilt, blue line) and using both FunTaxIS-lite (FT, orange line) and PANNZER (PZ, green line) taxonomic constraints. In panel B), the wFmax evaluation is reported for each subontology. In panel C), the Smin evaluation is reported for each subontology.*

# SUPPLEMENT 3: Comparison between automatic constraints (FunTaxIS-lite) and consortium constraints (GOC).

The quantification of taxonomic restrictions is documented in Supplementary Table 1. The dataset and the number of constraints generated refer to the GOA release date of 2023_05, Uniprot release date of 2023_03, GO release date of 2023_06, and Taxonomy release date of 2023_05. Taxonomic constraints were automatically generated by FunTaxIS-lite and those provided by GOC (Gene Ontology Consortium) have been reported separately. On average, FunTaxIS-lite's automatic restrictions covered 90% of GOC's restrictions, while the total number of restrictions generated solely by FunTaxIS-lite increased by an average of more than 300% compared to GOC's constraints. **Figure S3** displays the Venn diagram depicting the taxonomic constraints for the four species included in the benchmark.


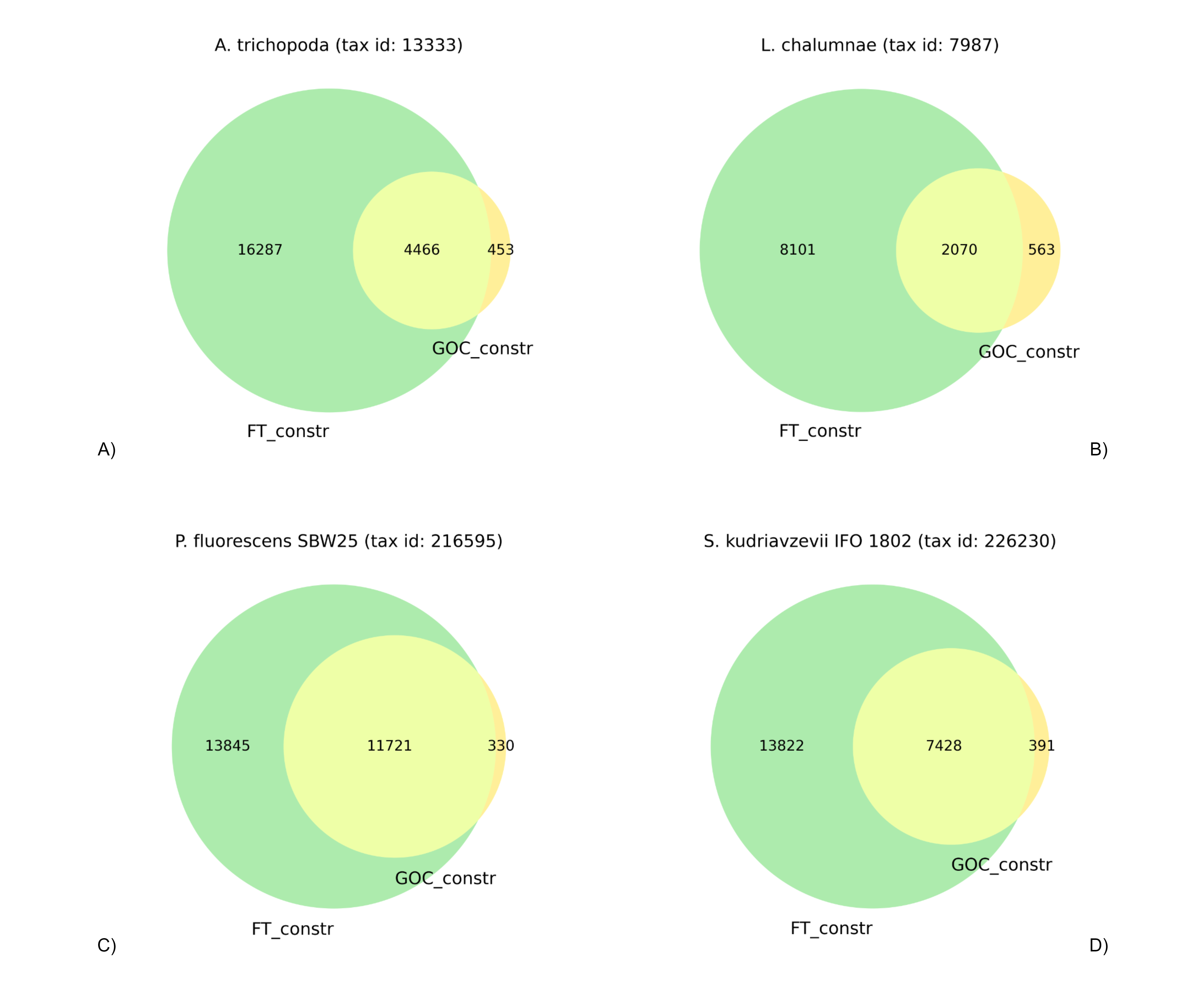


***Figure S3:*** *Venn diagrams showing the number of “automatic constraints” provided by FunTaxIS-lite and the number of “consortium constraints” provided by the Gene Ontology Consortium. The percentage of common constraints is more than 90% in A. trichopoda (A), P. fluorescens SBW25 (B) and S. kudriavzevii IFO 1802 (D) and more than 70% in L. chalumnae (B), calculated on the reference taxon nodes “Magnoliopsida”, “Pseudomonadales”, “Saccharomyces” and “Euteleostomi” respectively. The number of automatic, consortium and common constraints for all the other reference taxon nodes are reported in the* ***Suppl. Tab. 1****.*
